# The causal relationship between obesity and skin and soft tissue infections: A two-sample Mendelian randomization study

**DOI:** 10.3389/fendo.2022.996863

**Published:** 2022-12-07

**Authors:** Hongxin Hu, Jian Mei, Mei Lin, Xianwei Wu, Haibin Lin, Guoli Chen

**Affiliations:** ^1^ Department of Orthopedic Surgery, Affiliated Hospital of Putian University, Putian, China; ^2^ Department of Orthopaedic Surgery, Experimental Orthopaedics, Centre for Medical Biotechnology (ZMB/Biopark 1), University of Regensburg, Regensburg, Germany; ^3^ Department of Surgery, Affiliated Hospital of Putian University, Putian, China

**Keywords:** obesity, skin and soft tissue infections, genome-wide association study, Mendelian randomization, causal relationship

## Abstract

**Objective:**

Many observational studies have shown that obesity strongly affects skin and soft tissue infections (SSTIs). However, whether a causal genetic relationship exists between obesity and SSTIs is unclear.

**Methods:**

A two-sample Mendelian randomization (MR) study was used to explore whether obesity is causally associated with SSTIs using a publicly released genome-wide association study (GWAS). An inverse-variance weighted (IVW) analysis was used as the primary analysis, and the results are reported as the odds ratios (ORs). Heterogeneity was tested using Cochran’s Q test and the I^2^ statistic, and horizontal pleiotropy was tested using the MR−Egger intercept and MR pleiotropy residual sum and outlier (MR-PRESSO).

**Results:**

The results of the MR analysis showed a positive effect of BMI on SSTIs (OR 1.544, 95% CI 1.399-1.704, *P*= 5.86 × 10^-18^). After adjusting for the effect of type 2 diabetes (T2D) and peripheral vascular disease (PVD), the positive effect still existed. Then, we further assessed the effect of BMI on different types of SSTIs. The results showed that BMI caused an increased risk of impetigo, cutaneous abscess, furuncle and carbuncle, cellulitis, pilonidal cyst, and other local infections of skin and subcutaneous tissues, except for acute lymphadenitis. However, the associations disappeared after adjusting for the effect of T2D and PVD, and the associations between BMI and impetigo or cellulitis disappeared. Finally, we assessed the effects of several obesity-related characteristics on SSTIs. Waist circumference, hip circumference, body fat percentage, and whole-body fat mass, excluding waist-to-hip ratio, had a causal effect on an increased risk of SSTIs. However, the associations disappeared after adjusting for the effect of BMI.

**Conclusion:**

This study found that obesity had a positive causal effect on SSTIs. Reasonable weight control is a possible way to reduce the occurrence of SSTIs, especially in patients undergoing surgery.

## Introduction

Obesity is an excess of body fat that is detrimental to health and is often assessed clinically by the body mass index (BMI) (1). Obesity is a major public health problem, and in 2016, the World Health Organization (WHO) estimated that more than 1.9 billion people aged 18 years and older were overweight. More than 650 million of them are obese. In addition, more than 340 million children and adolescents aged 5-19 years are overweight or obese (2). Obesity reduces the health-related quality of life and longevity, and increases the risk of type 2 diabetes, coronary artery disease, gallbladder disease, and hyperlipidemia (3). Skin and soft tissue infections (SSTIs) are pathogenic bacteria that invade the epidermis, dermis, and subcutaneous tissues and induce a host response, and the diagnosis is based primarily on clinical features (4–6). The clinical features include sclerosis, erythema, fever, and pain or induration. Local manifestations may be accompanied by systemic signs and symptoms, such as fever, chills, and sometimes hemodynamic instability (5). SSTIs constitute the most common infectious disease in all age groups, and their incidence is increasing every year with the increase in surgical procedures, the use of immunosuppressive drugs, and cancer (7). SSTIs often require inpatient treatment and place a great burden on the health care system (8–10).

A cohort study involving 171,322 adults from 2011 to 2016 found that increased BMI was associated with an increased risk of cellulitis and hospitalization for cellulitis, and that obesity was an independent risk factor for cellulitis, after adjusting for confounders (11). Another case−control study noted that obese patients were 1.76 times more likely to have a surgical site infection than nonobese individuals (12). However, recently, it has been found that adipogenesis of the skin is an important source of resistance to infection and antimicrobial peptides (13, 14), which contradicts many observations regarding the increased risk of bacterial skin infections in obese individuals (15). Based on the results of these studies, the causal relationship between obesity and SSTIs is unclear as some confounding factors may have biased the results, such as the strong association between obesity and type 2 diabetes (T2D) and peripheral vascular disease (PVD) (5, 16). Therefore, the causal relationship between obesity and SSTIs has not been established.

Genetic epidemiology is used to elucidate the determinants of disease because the inheritance of genetic variants is random and cannot be confounded by additional risk factors (17, 18). Mendelian randomization is a method used to assess whether a causal relationship exists between exposure factors and outcomes using genetic variants as instrumental variables that are equally, randomly, and independently distributed during the split (19–21). Therefore, we used two-sample MR to assess the causal relationship between study obesity and SSTIs.

## Methods

### Study design

**Figure 1:**
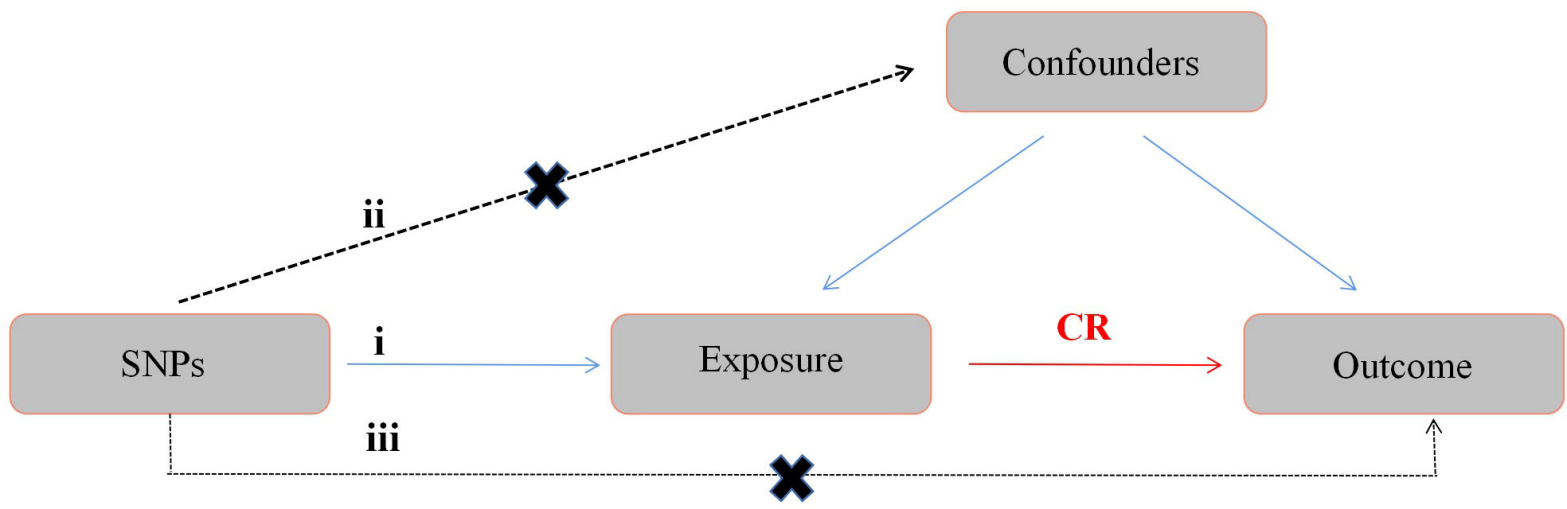
Diagram of the MR study design. The following three assumptions should be satisfied: 1) SNPs should be closely associated with exposure; 2) the selected SNPs should be independent of confounding factors; and 3) the SNPs affect the results only through the exposure factor. MR, Mendelian randomization; SNPs, single nucleotide polymorphisms; CR, causal relationship.

A two-sample MR design were used in this study, and genome-wide association study (GWAS) data were used to determine the causal relationship between obesity and SSTIs. Single nucleotide polymorphisms (SNPs) were instrumental variables for obesity. The BMI is often used to measure and assess obesity in the clinical practice (22). In addition, several other obesity-related characteristics were used in the assessment, including waist circumference (WC), hip circumference (HC), waist-to-hip ratio (WHR), body fat percentage (BF), and whole body fat mass (FM) (23–25). The main assumptions of this method include the following: 1) SNPs are associated with exposure factors, 2) SNPs are independent of confounding factors, and 3) SNPs affect outcomes only *via* exposure factors (**Figure 1**).

### Genome-wide association study summary data

The two-sample MR analysis in this study was performed based on data from a GWAS. BMI, obesity-related characteristics, and SSTIs (SSTIs as defined by the International Classification of Diseases- Tenth Revision code L00-L08) were obtained from the MRC IEU OpenGWAS database (https://gwas.mrcieu.ac.uk/), which consists mainly of GWAS data that are currently publicly available for use in Mendelian randomization analyses (26, 27). All GWAS were tested using imputed genotype data from the UK Biobank study. All GWASs were tested using imputed genotype data from the UK Biobank study. The GWAS summary of BMI was based on 461460 individuals of European ancestry, and the GWAS summary statistics of SSTIs were based on 218792 individuals in the European population (10343 SSTI cases and 208449 controls).

We analyzed the causal relationship between BMI and the risk of SSTIs using univariate MR. Next, we reassessed the effect of BMI on SSTIs after adjusting for the effect of T2D and PVD on BMI. Then, we assessed the effect of SSTI on BMI. We performed a stratified analysis of the different types of SSTIs to further assess the effect of BMI on different types of SSTIs. Finally, we assessed the causal effects of several obesity-related characteristics. The details of the data sources are provided in **Supplementary Table S1**.

### Instrumental variable selection

To select eligible genetic IVs that met the MR assumptions, we established a quality control technique. First, we selected independent SNPs that were strongly associated with the exposure factors with *p value*s < 5×10^-8^. In assessing the effect of SSTIs on BMI, we expanded the *p* value to < 1×10^-5^ to select suitable genetic instruments considering that few SNPs were associated with SSTIs at the *p* value < 5×10^-8^ level. Then, to exclude SNPs with strong linkage disequilibrium, we performed an aggregation process (R2<0.001) (28). Finally, to ensure that the effect alleles belonged to the same allele, we adjusted the exposure and outcome datasets to eliminate ambiguous SNPs with inconsistent alleles and SNPs with intermediate allele frequencies (29).

We calculated the *F* statistic of each SNP as follows: F=R2×(N-2)/(1-R2) (30). R2 denotes the variance of exposure explained by each IV according to the calculation method used in Papadimitriou et al. (30). IVs with F-statistic values less than 10 are considered weak instruments and were excluded from the MR analysis (31).

### Statistical analysis

The inverse variance weighted (IVW) method was used as the main method to analyze the causal relationship between obesity and SSTIs. The causal effect of each SNP on the outcome was assessed by calculating the Wald ratio of each SNP, and the inverse variance of the SNP was used as the weight in a meta-analysis to evaluate the combined causal effect. In addition, we used MR−Egger, weighted median, sample mode, and weighted mode to assess the causal relationship between BMI and SSTIs. MR−Egger has low statistical power; thus, the focus is more on direction and effect (32, 33). The weighted median provides a reliable Mendelian evaluation when 50% of the instrument variables are not valid (34). The odds ratio (OR) and 95% confidence interval (CI) were used to assess the relative risk.

We used the Cochran’s Q value and I^2^ statistic to assess heterogeneity among the SNPs and the MR−Egger method to test for horizontal pleiotropy. In addition, the MR pleiotropy residual sum and outlier (MR-PRESSO) method was used to detect outliers in the analysis and assess the adjusted causal effects after excluding the outliers (35). A sensitivity analysis was performed using the leave-one-out sensitivity test to assess the validity and stability of the MR results. We used MR-Steiger filtering to remove SNPs that implied reverse causal direction (36).

The data were analyzed using R (version 4.1.2) software with the R packages “two-sample MR” and “MRPRESSO”. *P*< 0.05 was considered statistically significant. The data used in this study were publicly available, and therefore, the study did not require ethical approval.

## Results

### Mendelian randomization to assess the causal relationship between obesity and SSTIs

**Figure 2:**
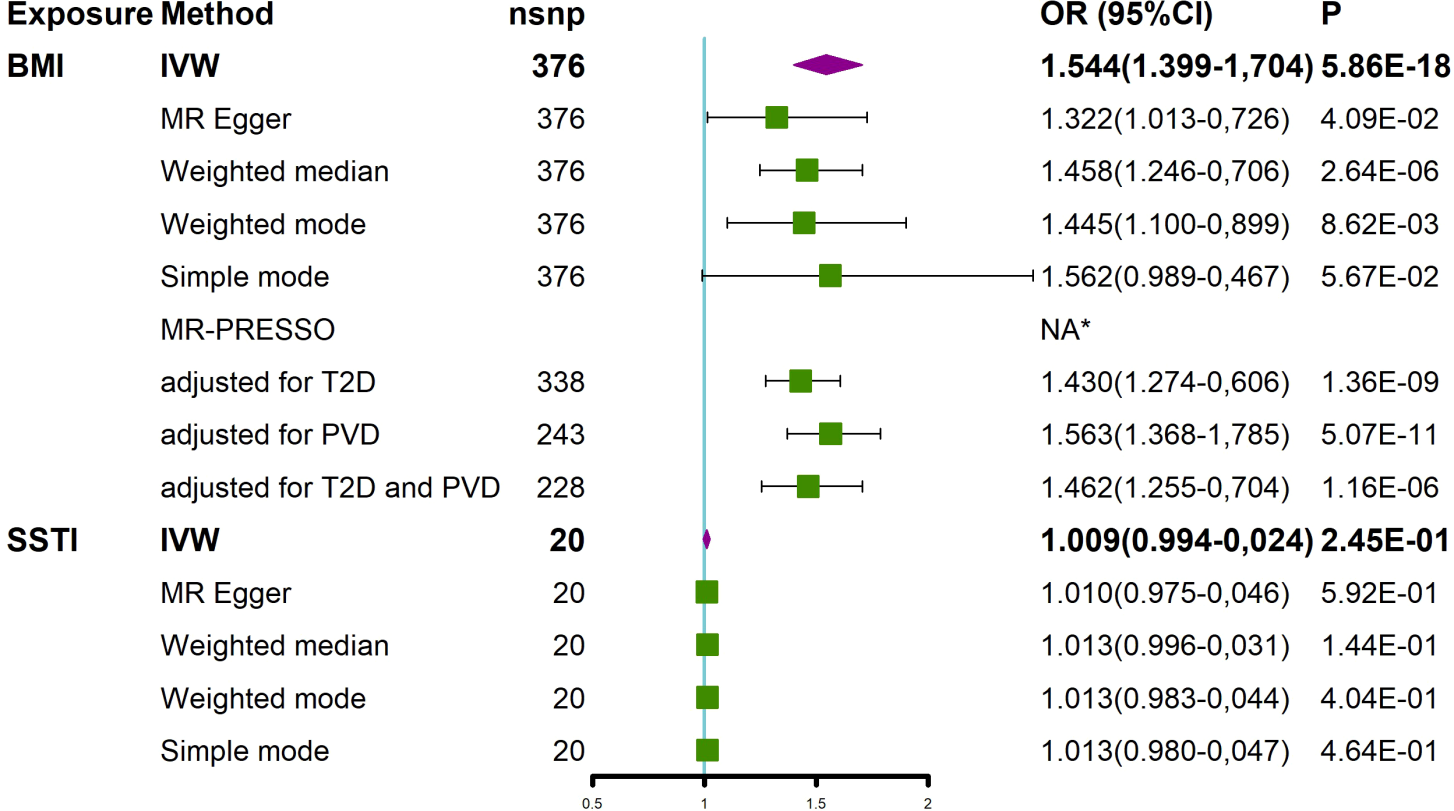
MR analysis showed the causal relationship between BMI and SSTIs. *No outliers were detected. BMI, body mass index; IVW, inverse variance weighted; nsnp, number of single nucleotide polymorphism; OR, odds ratio; CI, confidence interval; MR-PRESSO, MR Pleiotropy REsidual Sum and Outlier; T2D, type 2 diabetes; PVD, peripheral vascular disease; SSTI, skin and soft tissue infection.

The effect of BMI on the risk of an SSTI was first assessed using the univariate MR method (**Figure 2** and **Supplementary Figure S1**). The results using the IVW method showed that with a 1-SD increase in the BMI level, the OR of SSTIs was 1.544 (95% CI 1.399-1.704, *P*= 5.86 × 10^-18^). The MR−Egger method (OR=1.322, 95% CI 1.013-1.726, *P*= 4.09 ×10^-2^) and weighted median (OR=1.458, 95% CI 1.246-1.706, P=2.64 × 10^-6^) also showed consistent results. In the above analysis, Cochran’s Q and I^2^ tests did not detect heterogeneity, and the MR−Egger test did not detect horizontal pleiotropy (**Supplementary Figure S2** and **Supplementary Table S2**). The MR-PESSO method did not detect outliers; thus, the association between BMI and SSTIs and its significance remained unchanged (**Figure 2**). To assess whether these results were affected by a single SNP, we performed the leave-one-out sensitivity test, which showed that the causal effect of BMI on SSTIs did not significantly fluctuate in the absence of any single SNP (**Supplementary Figure S3**). We performed MR-Steiger filtering of the SNPs of BMI and did not find SNPs with reverse causality.

Next, we assessed the effect of an adjusted BMI on SSTIs after excluding T2D and PVD, which often coexist with obesity (**Figure 2**). After adjusting for T2D, BMI was still causally associated with an increased risk of SSTIs (OR=1.430, 95% CI 1.274-1.606, P=1.36×10^-9^). After adjusting for PVD, BMI was also causally associated with an increased risk of SSTIs (OR=1.563, 95% CI 1.368-1.785, P=5.07×10^-11^). Finally, after we adjusted for T2D and PVD, an increase in BMI was still positively associated with an increased risk of SSTIs (OR=1.462, 95% CI 1.255-1.704, P=1.16×10^-06^).

Finally, we also explored whether SSTIs had a causal effect on the BMI. The results of the IVW method showed no effect of SSTIs on the BMI. Due to the presence of heterogeneity (**Supplementary Table S2**), the results of the weighted median analysis were used according to Nazarzadeh et al. (37). The results of the weighted median method showed no effect of SSTIs on BMI (**Figure 2**).

### Causal effects of BMI on different types of SSTIs

**Figure 3:**
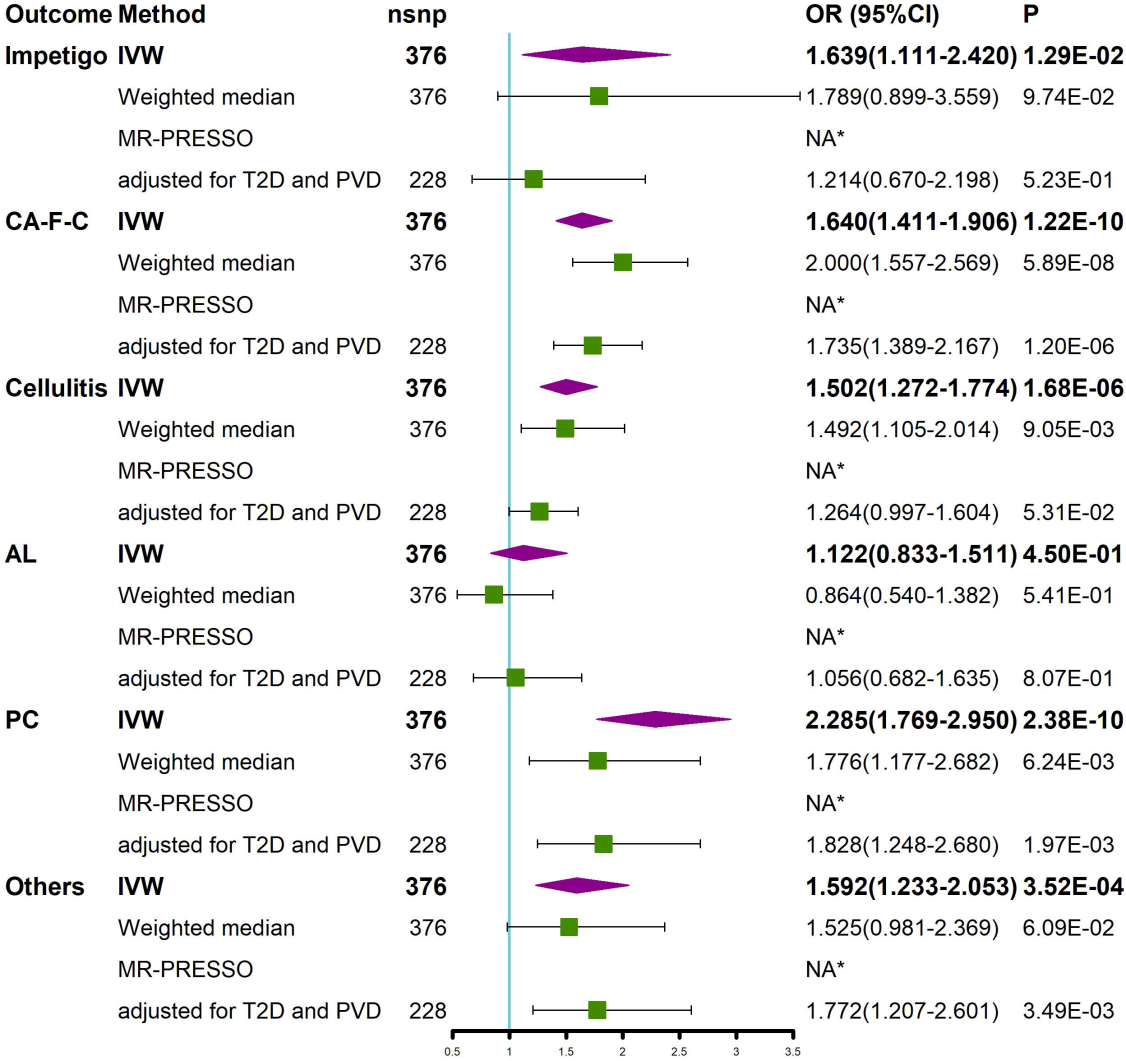
Univariable MR and multivariable MR analyses showed the causal relationship between BMI and different types of SSTIs. Univariable MR was performed using the IVW, weighted median, and MR-PRESSO methods. Multivariable MR was performed to assess the effect of BMI on different types of SSTIs after adjusting for T2D and PVD. *No outliers were detected. BMI, body mass index; IVW, inverse variance weighted; nsnp, number of single nucleotide polymorphism; OR, odds ratio; CI, confidence interval; MR-PRESSO, MR Pleiotropy REsidual Sum and Outlier; T2D, type 2 diabetes; PVD, peripheral vascular disease; CA-F-C, cutaneous abscess, furuncle and carbuncle; AL, acute lymphadenitis; PC, pilonidal cyst; Others, other local infections of skin and subcutaneous tissues; SSTIs, skin and soft tissue infections.

We performed a stratified analysis of different types of SSTIs (according to ICD-Tenth Revision) to further assess the effect of BMI on different types of SSTIs (**Figure 3**). SNPs associated with staphylococcal scalded skin syndrome (SSSS) were not available in the GWAS; thus, SSSS was not included in the type stratification of SSTIs. The results of the univariate MR analysis showed that an increase in BMI caused an increased risk of impetigo, cutaneous abscess, furuncle and carbuncle (CA-F-C), cellulitis, pilonidal cyst (PC), and other local infections of skin and subcutaneous tissues, except for acute lymphadenitis (AL) (**Figure 3**). Next, we adjusted for the effects of T2D and PVD. The results of the multivariate MR study showed that after adjusting for both T2D and PVD, an increase in BMI resulted in an increased risk of CA-F-C, PC, and other local infections of skin and subcutaneous tissues (**Figure 3**). In addition, no outliers of BMI were detected by the MR-PESSO method (**Figure 3**).

### Causal effect of obesity-related characteristics on SSTIs

**Figure 4:**
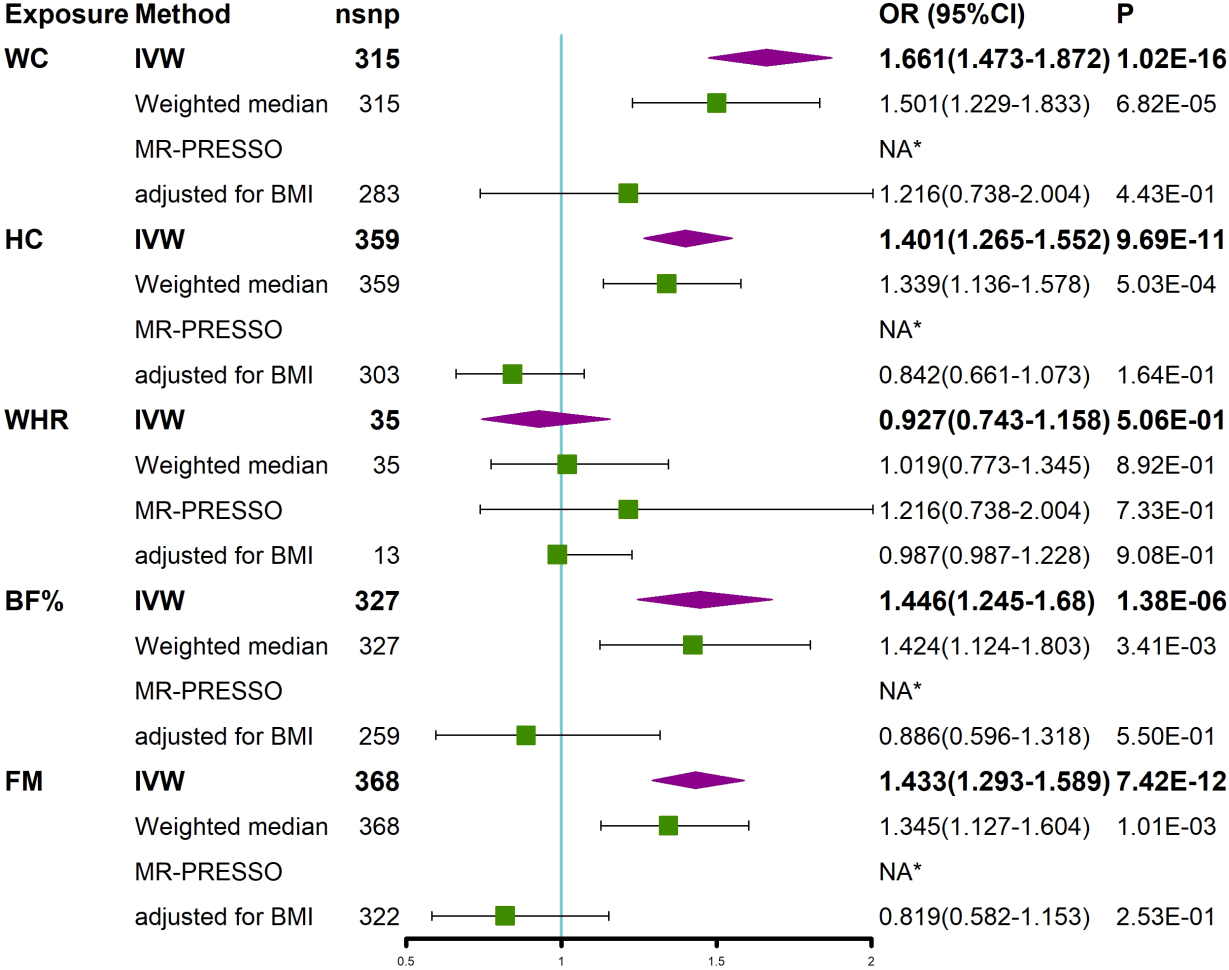
MR analyses showed a causal relationship between obesity-related characteristics and SSTIs. Univariable MR was performed using the IVW, weighted median, and MR-PRESSO methods. Multivariable MR was performed to assess the effect of obesity-related characteristics on SSTIs after adjusting for BMI. *No outliers were detected. BMI, body mass index; IVW, inverse variance weighted; nsnp, number of single nucleotide polymorphism; OR, odds ratio; CI, confidence interval; MR-PRESSO, MR Pleiotropy REsidual Sum and Outlier; WC, waist circumference; HC, hip circumference; WHR, waist-to-hip ratio; BF, body fat percentage, FM, whole body fat mass; SSTIs, skin and soft tissue infections.

We investigated whether obesity-related characteristics (WC, HC, WHR, BF, and FM) had a causal effect on SSTIs. The univariate MR analysis showed that the obesity-related characteristics of WC, HC, BF, and FM, excluding WHR, had a causal effect on the increased risk of SSTIs (**Figure 4**). The correlations were consistent in all sensitivity analyses, although the MR−Egger regression analysis detected pleiotropy in HC and BF. After removing outliers using the MR-PRESSO method, these factors remained significantly associated with SSTIs (**Figure 4**). Multivariate MR analyses adjusting for BMI were performed because these obesity-related characteristics were highly correlated with BMI. The results of the study showed that none of these obesity-related features were significantly associated with SSTIs after adjusting for BMI (**Figure 4**).

## Discussion

Many previous clinical studies have noted that obesity is a risk factor for a variety of diseases, including SSTIs. However, due to the limitations of previous studies, these findings may be influenced by confounding factors and reverse causality. Our study was based on MR to explore the relationship between obesity and SSTIs. The results of the study found a positive causal effect of obesity on an increased risk of SSTIs. This positive causality persisted after adjusting for the effects of T2D and PVD.

Our findings suggest that obesity increases the risk of SSTIs, which is similar to the results of previous studies (15). This finding may be related to the metabolic effects of obesity and inadequate blood supply to adipose tissue (AT), which leads to poor host immune recruitment and thus causes skin defenses to be diminished (9, 38). Healthy AT acts as a defense barrier against microorganisms. Adipocytes are able to produce antimicrobial peptides (AMPs) in response to skin infections, thereby strengthening the innate immune defenses of the skin (39). Several findings confirm that host defense is mediated through adipocyte production of AMPs and that the inhibition of adipocytes reduces the expression of AMPs (13, 14). Recently, an animal study (40) found that obesity in mice leads to a loss of dermal preadipocytes (pADs) and inhibits the ability to initiate responsive adipose expression of antimicrobial peptides, and obesity leads to a loss of dermal fibroblasts (dFBs), which have the ability to polarize into mature adipocytes capable of expressing antimicrobial peptides, further promoting the development of infection. Obesity causes hypertrophy and proliferation in adipocytes, leading to a rapid increase in adipose tissue, and when angiogenesis cannot match the expanded AT, local hypoxia in AT causes an increase in pro-inflammatory leptin and a decrease in anti-inflammatory adiponectin (41–44). In addition, hypoxia progresses to induce endoplasmic reticulum stress, which combined with excess extracellular matrix (ECM) deposition, causes AT fibrosis (41, 45). All these changes promote the release of inflammatory factors and intensify the local inflammatory response, ultimately leading to adipocyte dysfunction and metabolic changes and causing decreased immunity and insulin resistance (41, 43–46). Furthermore, hypoxia in AT causes adipocytes to undergo anaerobic glycolysis, promoting the increased production and release of lactate, which further promotes the inflammatory pathway of macrophages, resulting in chronic inflammation (47, 48).

In chronic inflammation, immune cells are constantly stimulated by cytokines or chemokines and are activated, eventually causing immune cell depletion (49). In addition, chronic inflammation decreases the formation of memory T cells, reducing the ability of T cells to protect the host against infection (50). Recently, it has been found that long-term stimulation by chronic inflammation causes a significant decrease in the proliferative capacity of hematopoietic stem cells, the origin of immune cells, resulting in accumulative and irreversible functional damage and long-term suppressive effects on hematopoiesis, further contributing to immune cell failure and decreased immune function (51).

Obesity also affects the function of immune cells, which leads to decreased immune function and promotes the development of infections. Macrophages are key mediators of inflammation in adipose tissue and are the most abundant immune cells (52). In the obese state, macrophages mainly accumulate around adipocytes, forming crown-like structures and proliferating to clear dysfunctional and necrotic adipocytes, and inflammation cannot be cleared due to persistent obesity; then, inflammation changes from local inflammation to a systemic chronic inflammatory state (41, 53). Other research has found that macrophages exhibit an anti-inflammatory M2 phenotype in thin mice, whereas in diet-induced obese (DIO) mice, macrophages switch from an anti-inflammatory M2 phenotype to a proinflammatory M1 phenotype, leading to chronic inflammation (54, 55). Additionally, these macrophages show decreased phagocytosis and a decreased ability to clear bacteria (44). In addition, obesity also inhibits T-lymphocyte receptor diversity, leading to abnormalities in the antigen presentation process, decreasing the efficiency of T-lymphocyte responses and increasing susceptibility to infection in obese individuals (44, 56). Neutrophils play a role in the inflammatory response induced by obesity, and neutrophils in AT produce chemokines and cytokines that promote macrophage infiltration, contribute to chronic low-grade inflammation, and induce insulin resistance (57). Furthermore, the imbalance of neutrophil elastase (NE) and its inhibitor α1-antiprotease in obese individuals causes chronic inflammation and insulin resistance (58). Moreover, obesity causes the downregulation of neutrophil function, which reduces bacterial clearance (44, 59). Furthermore, the humoral immune response is defective in obese individuals, and B lymphocytes in obese individuals express a proinflammatory phenotype that reduces their ability to optimally respond to infection (44).

Obesity or high-fat diets modify the composition of the gut microbiota and increase intestinal permeability, leading to the passage of bacteria and bacterial products (e.g., lipopolysaccharides, LPS) across the intestinal barrier into the circulation, which can activate innate immune cells and immune signaling pathways and contribute to inflammation formation (2, 44, 60–63). In addition, a high-fat diet increases the proportion of gram-negative bacteria, further promoting the absorption of LPS across the intestinal barrier (60, 64). These factors all lead to a low-grade systemic inflammatory response (65–67). As obesity advances, the eventual mild low-grade inflammation becomes chronic inflammation that further causes systemic inflammation (68–70).

Overall, obesity leads to immune system dysregulation, a reduced cell-mediated immune response, and modified gut microbiota, representing possible reasons for the increased risk of infection associated with obesity (16, 38, 71–73). Similar to the findings reported by Conway et al. (74), the present study also showed that the genetic prediction of BMI was positively causally associated with an increased risk of SSTIs. Therefore, the risk of infection should be noted in obese patients, especially those who undergo surgery. Preoperatively, obese patients may be advised to lose weight to reduce systemic inflammation and the risk of infection (75).

We performed a stratified analysis of the types of SSTIs, and the findings showed that an increased BMI was positively associated with an increased risk of impetigo, CA-F-C, cellulitis, PC, and other local infections of skin and subcutaneous tissues. However, after adjusting for T2D and PVD, an increased BMI was not significantly associated with the development of cellulitis, which may be due to obesity-induced elevated blood glucose due to insulin resistance and PVD to further mediate the development of cellulitis (76, 77). In addition, although we stratified the degree of severity and site of SSTIs, the GWAS lacked data of the different degrees or different sites of SSTIs; thus, an MR analysis with stratification of different degrees or sites of SSTIs is not available.

In addition, this study evaluated the relationship between several other obesity-related characteristics and the risk of SSTIs. Previous studies have found that obesity characteristics, such as WC and BF, are predictors of infection (78, 79). In the univariate MR analysis, we also found a positive association between waist circumference, hip circumference, body fat percentage and whole-body fat mass and the risk of SSTIs. However, this association may be mediated through the effect of BMI as we did not observe this significant effect after adjusting for BMI. Furthermore, the OR of HC, BF and FM on SSTIs was less than 1 after adjusting for BMI, although there was no significance, possibly implying that increased HC, BF, and FM are protective factors against SSTIs, similar to a recent cohort study that found a lower risk of death in people with a small WC and large HC (80). However, our findings are only based on the MR method, and large clinical studies are still needed to confirm whether such a relationship exists. In view of our findings, we recommend using BMI rather than other obesity characteristics in the risk assessment of SSTIs in the clinic.

To the best of our knowledge, this study is the first to use a two-sample MR analysis to assess the causal relationship between obesity and SSTIs. In addition, the genetic variants used as IVs were extracted from the largest GWAS. However, there are some limitations to this study. 1). The MR analysis conducted in this study was based on a European cohort, and whether there is a demographic effect on the results is unclear. 2). The genetic data of exposure and outcome were pooled data from a GWAS, and relevant data, such as disease prevalence and age specificity, are lacking. 3). This study focuses on the causal relationship between obesity and SSTIs, but whether a causal relationship exists between weight loss and SSTIs is unclear.

## Conclusion

This study shows that a positive causal relationship exists between a genetically predicted increase in BMI and an increased risk of SSTIs, further deepening our understanding of obesity causing SSTIs and providing guidance for prevention and treatment.

## Data availability statement

The datasets presented in this study can be found in online repositories. The names of the repository/repositories and accession number(s) can be found below: https://gwas.mrcieu.ac.uk/datasets/.

## Author contributions

HH and ML performed the study and wrote the manuscript. XW and HL revised the manuscript. JM wrote sections of the manuscript and performed the statistical analysis. GC designed the study. All authors contributed to the article and approved the submitted version.

## Supplementary material

Supplementary Figure 1 Plots of MR estimates of the causal relationship between SSTIs and BMI. **(A)** Scatter plot of SNPs showing the causal effect of BMI on SSTIs. The log odds ratio of risk is demonstrated, and five different methods were used. **(B)** Forest plot of SNPs associated with BMI on SSTIs. MR, Mendelian randomization; SSTI, skin and soft tissue infection; BMI, body mass index; SNP, single nucleotide polymorphism.

Supplementary Figure 2 Funnel plot showed there were no significant heterogeneity among SNPs. SNPs, single nucleotide polymorphisms.

Supplementary Figure 3 Leave-one-out analysis plots for BMI on SSTIs. MR, Mendelian randomization; SSTI, skin and soft tissue infection; BMI, body mass index.

Supplementary Table 1 Data source information. BMI, body mass index; T2D, type 2 diabetes; SSTI, skin and soft tissue infection.

Supplementary Table 2 Heterogeneity tests and directional horizontal pleiotropy test.
