# Supplementary figures and images for: The causal relationship between obesity and skin and soft tissue infections: A two-sample Mendelian randomization study
Source: Front Endocrinol (Lausanne). 2022 Dec 7;13:996863. doi: 10.3389/fendo.2022.996863 (PMC9768473; doi:10.3389/fendo.2022.996863)

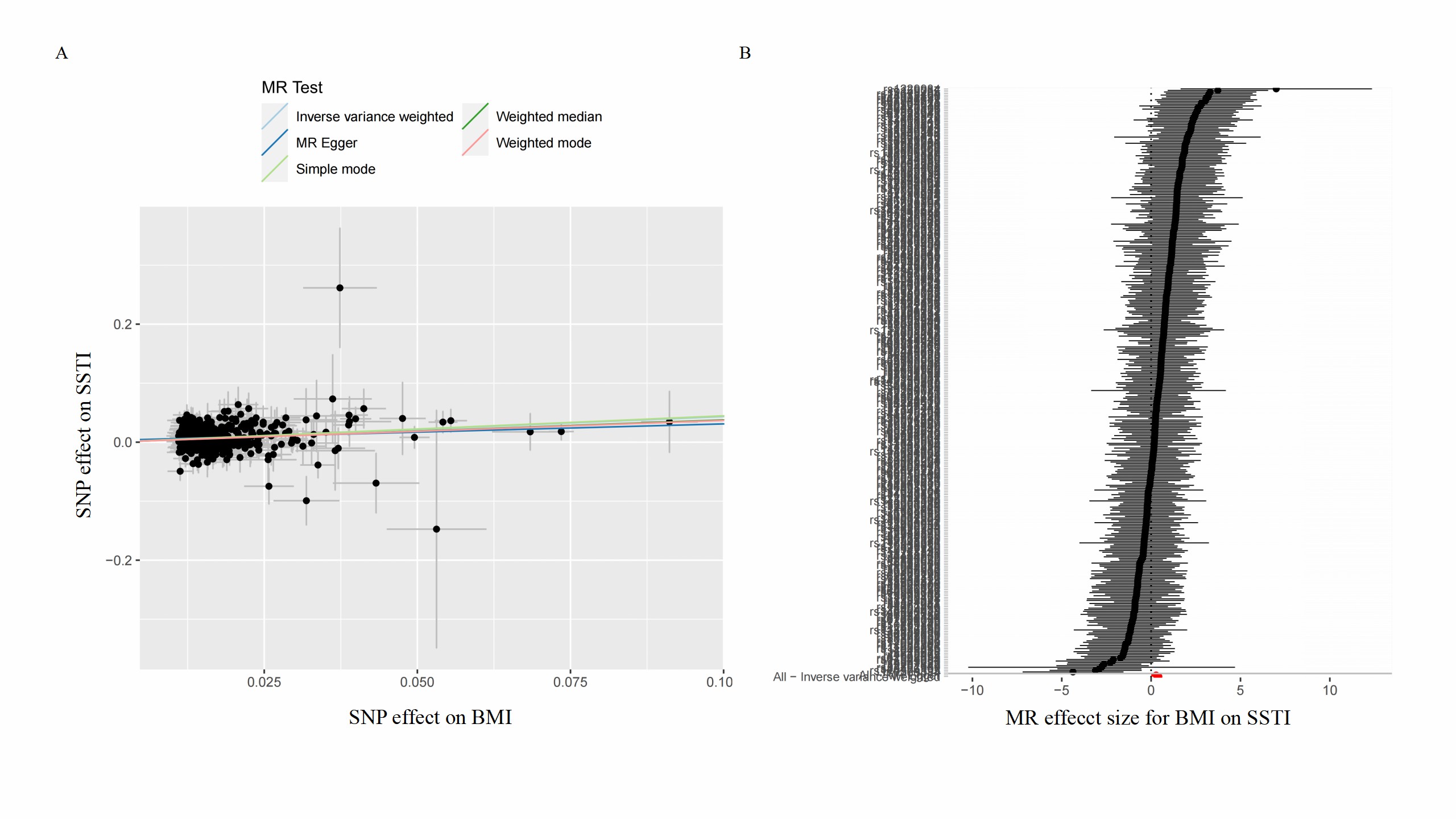

Supplement: Supplementary Figure 1 — Plots of MR estimates of the causal relationship between SSTIs and BMI. (A) Scatter plot of SNPs showing the causal effect of BMI on SSTIs. The log odds ratio of risk is demonstrated, and five different methods were used. (B) Forest plot of SNPs associated with BMI on SSTIs. MR, Mendelian randomization; SSTI, skin and soft tissue infection; BMI, body mass index; SNP, single nucleotide polymorphism. [file Image_1.jpg]

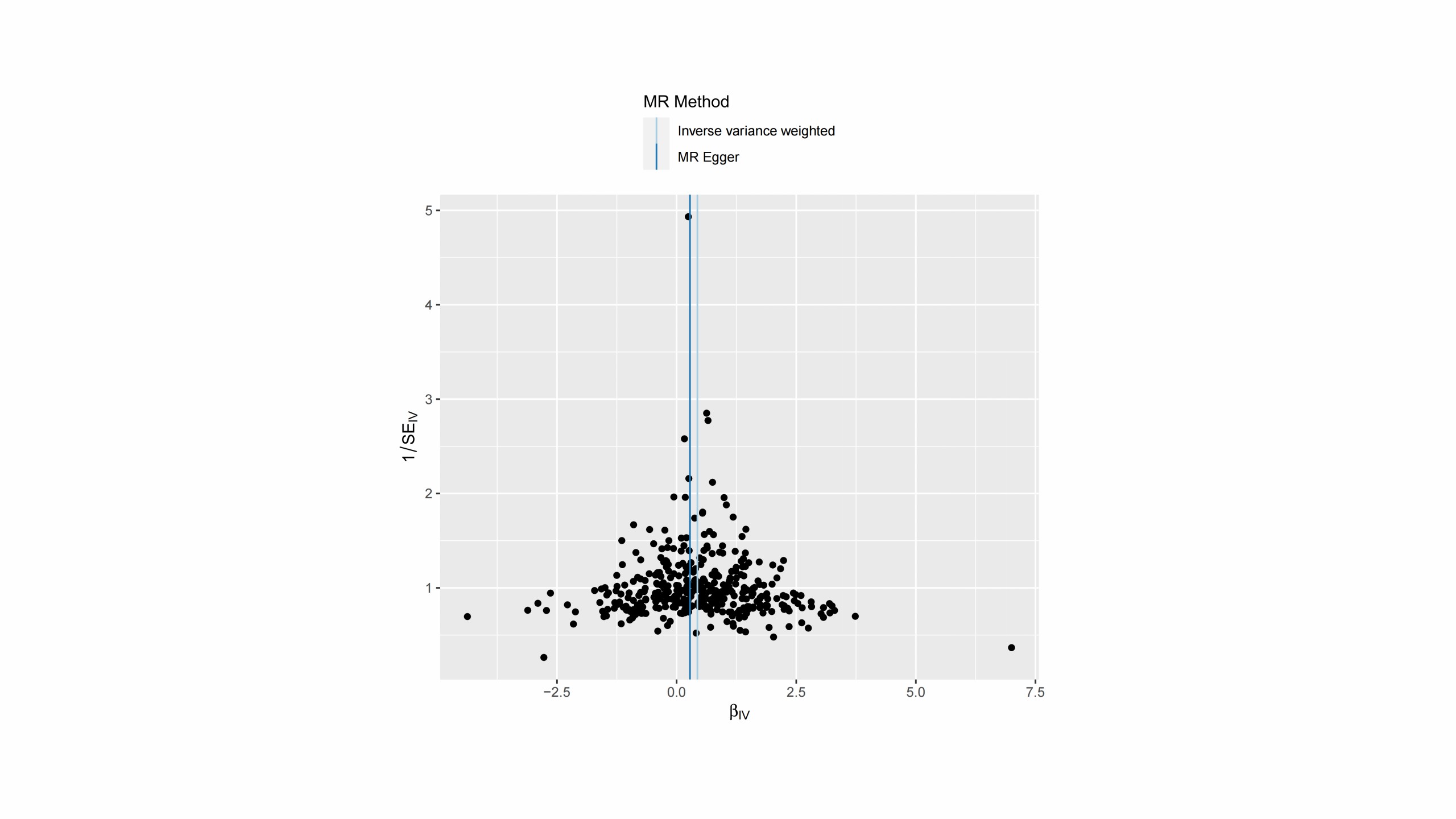

Supplement: Supplementary Figure 2 — Funnel plot showed there were no significant heterogeneity among SNPs. SNPs, single nucleotide polymorphisms. [file Image_2.jpg]

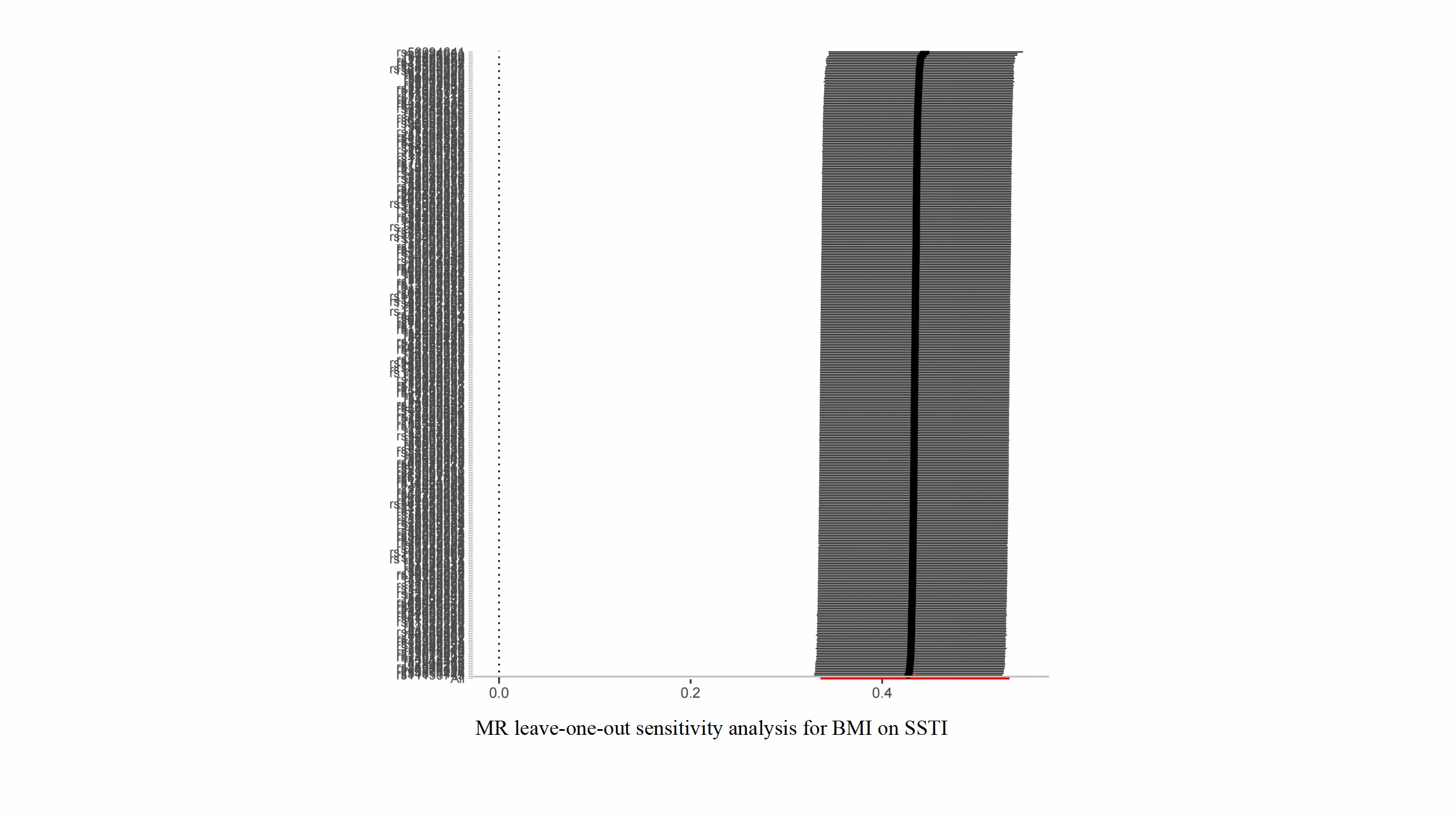

Supplement: Supplementary Figure 3 — Leave-one-out analysis plots for BMI on SSTIs. MR, Mendelian randomization; SSTI, skin and soft tissue infection; BMI, body mass index. [file Image_3.jpg]
