# Supplementary material for: The causal relationship between obesity and skin and soft tissue infections: A two-sample Mendelian randomization study
Source: Front Endocrinol (Lausanne). 2022 Dec 7;13:996863. doi: 10.3389/fendo.2022.996863 (PMC9768473; doi:10.3389/fendo.2022.996863)
Supplement: Supplementary Table 2 — Heterogeneity tests and directional horizontal pleiotropy test. [file Table_2.docx]

**Supplementary Table 2. The Heterogeneity tests and Directional horizontal pleiotropy test.**

| **Exposure** | **Outcome** | **Methods** | **Cochran’s Q (*P*-value)** | **I^2^(%)** | **MR-Egger intercept (*P*-value)** |
| --- | --- | --- | --- | --- | --- |
| BMI | SSTI | MR Egger | 408.716 (0.105) | 8.5 | 0.003 (0.222) |
|  |  | Inverse variance weighted | 410.353 (0.101) | 8.6 |  |
| SSTI | BMI | MR Egger | 31.744 (0.033) | 0.40 | 0.0004 (0.835) |
|  |  | Inverse variance weighted | 31.819 (0.045) | 0.37 |  |
| BMI | Impetigo | MR Egger | 410.177 (0.096) | 8.8 | -0.012 (0.218) |
|  |  | Inverse variance weighted | 411.850 (0.092) | 8.9 |  |
| BMI | CA-F-C | MR Egger | 377.722 (0.436) | 1.0 | -0.005 (0.900) |
|  |  | Inverse variance weighted | 377.738 (0.451) | 0.7 |  |
| BMI | Cellulitis | MR Egger | 327.349 (0.961) | -0.14 | -0.002 (0.656) |
|  |  | Inverse variance weighted | 327.548 (0.963) | -0.14 |  |
| BMI | AL | MR Egger | 393.091 (0.239) | 4.9 | 0.021 (0.005) |
|  |  | Inverse variance weighted | 401.483 (0.166) | 6.6 |  |
| BMI | PC | MR Egger | 417.357 (0.060) | 10.4 | 0.007 (0.274) |
|  |  | Inverse variance weighted | 418.693 (0.059) | 10.4 |  |
| BMI | Other | MR Egger | 356.180 (0.738) | -0.05 | -0.003 (0.634) |
|  |  | Inverse variance weighted | 356.406 (0.747) | -0.05 |  |
| HC | SSTI | MR Egger | 406.773 (0.035) | 12.2 | 0.005 (0.058) |
|  |  | Inverse variance weighted | 410.894 (0.027) | 12.9 |  |
| WC | SSTI | MR Egger | 321.243 (0.362) | 2.6 | 0.001 (0.614) |
|  |  | Inverse variance weighted | 321.504 (0.373) | 2.3 |  |
| WHR | SSTI | MR Egger | 36.303 (0.317) | 9.0 | 0.066 (0.090) |
|  |  | Inverse variance weighted | 39.670 (0.232) | 14.3 |  |
| BF | SSTI | MR Egger | 394..946 (0.005) | 17.7 | 0.0009 (0.003) |
|  |  | Inverse variance weighted | 395.030 (0.005) | 17.5 |  |
| FM | SSTI | MR Egger | 406.401 (0.071) | 9.9 | 0.0002 (0.940) |
|  |  | Inverse variance weighted | 406.408 (0.076) | 9.7 |  |
